# The Biomolecular Basis of Adipogenic Differentiation of Adipose-Derived Stem Cells

**DOI:** 10.3390/ijms15046517

**Published:** 2014-04-16

**Authors:** Maria Giovanna Scioli, Alessandra Bielli, Pietro Gentile, Donatella Mazzaglia, Valerio Cervelli, Augusto Orlandi

**Affiliations:** 1 Institute of Anatomic Pathology, Department of Biomedicine and Prevention, Tor Vergata University of Rome, Via Montpellier, 00133 Rome, Italy; E-Mails: scioli@med.uniroma2.it (M.G.S.); alessandrabielli@hotmail.it (A.B.); donatella.mazzaglia@hotmail.it (D.M.); 2 Institute of Plastic Surgery, Department of Biomedicine and Prevention, Tor Vergata University of Rome, Via Montpellier, 00133 Rome, Italy; E-Mails: pietrogentile2004@libero.it (P.G.); valeriocervelli@virgilio.it (V.C.)

**Keywords:** adipose-derived stem cells, receptor tyrosine kinases, Akt, Erk-1, proliferation, differentiation, growth factors

## Abstract

There is considerable attention regarding the role of receptor signaling and downstream-regulated mediators in the homeostasis of adipocytes, but less information is available concerning adipose-derived stem cell (ASC) biology. Recent studies revealed that the pathways regulating ASC differentiation involve the activity of receptor tyrosine kinases (RTKs), including fibroblast growth factor, vascular endothelial growth factor, ErbB receptors and the downstream-regulated serine/threonine protein kinase B (Akt) and phosphatase and tensin homolog (PTEN) activity. RTKs are cell surface receptors that represent key regulators of cellular homeostasis but also play a critical role in the progression of cancer. Many of the metabolic effects and other consequences of activated RTKs are mediated by the modulation of Akt and extracellular signal-regulated protein kinases 1 (Erk-1) signaling. Akt activity sustains survival and the adipogenic differentiation of ASCs, whereas Erk-1 appears downregulated. The inhibition of FGFR-1, EGFR and ErbB2 reduced proliferation, but only FGFR-1 inihibition reduced Akt activity and adipogenesis. Adipogenesis and neovascularization are also chronologically and spatially coupled processes and RTK activation and downstream targets are also involved in ASC-mediated angiogenesis. The potentiality of ASCs and the possibility to modulate specific molecular pathways underlying ASC biological processes and, in particular, those shared with cancer cells, offer new exciting strategies in the field of regenerative medicine.

## 1. Introduction

**Figure 1.**
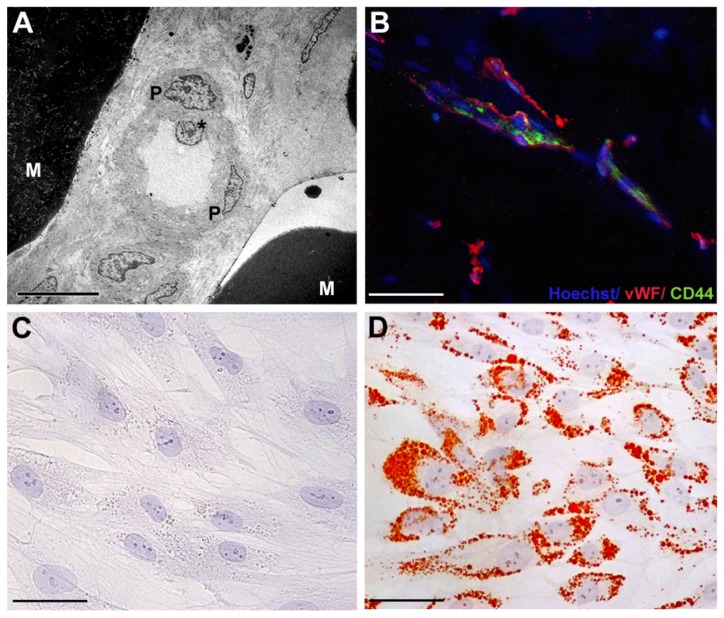
Human adipose-derived stem cells: origin, morphology and differentiation potential. (**A**) Transmission electron microscopy image of human adipose tissue, with large accumulation of electron dense intracellular lipid in mature adipocytes (M), and perivascular cells featuring ASCs (P) surrounding a small blood vessel with a prominent endothelial cell (*). Scale bar, 10 μm; (**B**) Merged double immunofluorescence of a cryostatic section of human adipose tissue showing CD44 positive ASCs (green) around von Willebrand factor (vWF, red) positive endothelial cells of a small vessel. Nuclei are stained with Hoechst (blue). Scale bar, 100 μm; Contrast phase images of Oil Red O negative staining (**C**) of serum-cultured ASCs and (**D**) strongly positive ASCs after combined platelet-derived growth factors plus insulin treatment showing abundant intracytoplasmic lipid droplets. Scale bar, 100 μm.

Despite the large attention regarding the role of receptor signaling and downstream pathways in cancer cells and cancer stem cells, less information concerning resident adult stem cell biology, in particular adipose-derived stem cells (ASCs), is available. Adipose tissue has been demonstrated to contain progenitor cells with a perivascular origin [1], as illustrated in Figure 1A,B. ASCs are able to differentiate in multiple cell lineages [2]. The potential plasticity and therapeutic utility of ASCs isolated from adult adipose tissue have been diffusely highlighted. The potentiality of ASCs offers new approaches in regenerative medicine and surgery for tissue and organ function reconstructions [3,4]. Therefore, it is relevant to better understand the molecular mechanisms underlying ASC biological processes and, in particular, those shared with cancer cells. In this review, we focused the attention on biomolecular basis of adipogenic differentiation of human ASCs.

## 2. Adipose-Derived Stem Cells: Phenotypic Characterization

Adult adipose tissue is a multifunctional organ that contains various cellular types, including mature adipocytes, macrophages, endothelial cells, smooth muscle cells, and preadipocytes, supported by connective tissue surrounding fine capillaries [1]. Preadipocytes, also known as adipose-derived stem cells (ASCs), have the potential to form bone, cartilage, muscle, and fat tissue. ASCs can be easily isolated from lipoaspirated subcutaneous adult adipose tissue by enzymatic digestion [2]. After centrifugation, a heterogeneous mixture of endothelial cells, smooth muscle cells, fibroblast, pericytes, mast cells and preadipocytes is obtained, named stromal vascular fraction (SVF). ASCs can be separated from SVF by adhesion to a plastic dish [2]. Before the discovery of the plasticity of ASCs, bone marrow was clinically considered the major tissue source of human adult stem cells, the so-called mesenchymal stem cells (MSCs) [2]. ASCs and MSCs share the ability to differentiate along multiple lineage pathways [2], without any risk of teratogenesis, as reported for human embryonic stem cells [5,6]. However, some characteristics of ASCs, in particular the maintenance of proliferating ability in culture, are even greater than those of MSCs [7]. The surface antigen profile of ASCs isolated from human adipose tissue, changes *in vitro* as a function of time and/or passage in culture [8]. After two or more passages *in vitro*, ASC surface immunophenotype resembles that of MSCs, with a similarity greater than 90% [2]. ASCs display stromal markers, such as CD44, CD90, CD73, CD166, and CD29 [2], pericytic markers, such as CD140a and CD140b, and cytoskeletal markers, such as α–smooth muscle actin and vimentin [1]. Nevertheless, some differences in surface protein expression have been described. The presence of the glycoprotein CD34 on the surface of human ASCs is not reported in MSCs [9].

## 3. The Receptor Tyrosine Kinase Family

**Figure 2.**
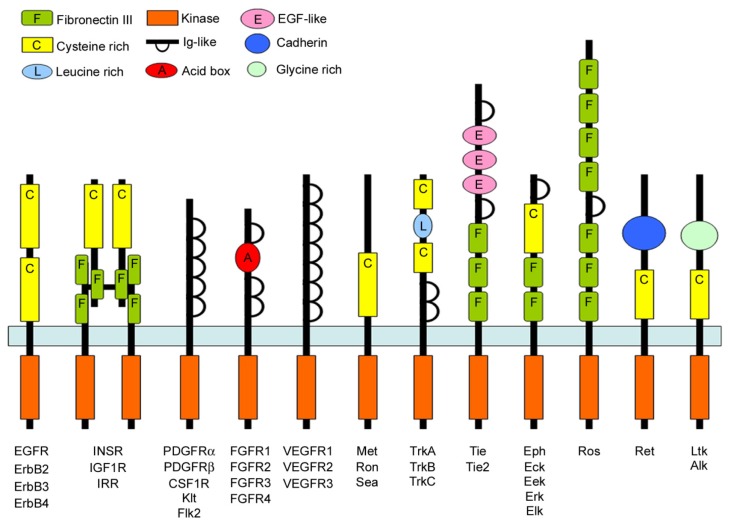
Receptor tyrosine kinase family. Schematic representation of receptor tyrosine kinase family involved in the regulation of biological properties and differentiation of ASCs.

Receptor tyrosine kinases (RTKs) are high-affinity cell surface receptors for several polypeptides, growth factors, cytokines, and hormones [10] (Figure 2). In humans, 58 different RTKs have been recognized, which fall into 20 subfamilies [10]. All RTKs, including vascular endothelial growth factor receptors (VEGFRs), epidermal growth factor receptor family (ErbBs), fibroblast growth factor receptors (FGFRs), platelet derived growth factor receptors (PDGFRs), insulin-like growth factor 1 receptor (IGF-1R), insulin receptor, have a similar molecular architecture, with ligand binding domains in the extracellular region, a single transmembrane helix, a cytoplasmic region that contains the protein tyrosine kinase (TK) domain plus additional carboxy (*C*-) terminal and juxtamembrane regulatory regions [10]. RTKs have assumed a key regulator role of many cellular processes, including proliferation, migration, differentiation, and survival [11,12]. RTKs play also a critical role in the development and progression of cancer [13]. Rapidly increasing knowledge of the cascade of biochemical events triggered by ligand stimulation of RTKs provides further evidence of the importance of their signaling pathways. RTKs play an integral role in intracellular signaling by both recruiting various proteins to specific locations and assembling particular networks of proteins [13]. Following ligand binding, receptor dimerization, and autophosphorylation, Src homology 2 (SH2) domain-containing proteins are recruited to phosphorylated tyrosine residues of the receptor [14]. These SH2 domain-containing proteins include the p85 component of the phosphoinositide 3-kinase (PI3K) pathway, phospholipase C-7 of the protein kinase C pathway, Src family kinase and p120-GAP, Shc, and Grb2 in the Ras pathway [15]. The principal downstream effector of PI3K is the serine/threonine protein kinase B (Akt), that is one of the most relevant actors in the biomolecular scenario of cell survival [14]. In mammals, up to now three genes have been identified encoding for different Akt isoforms. Akt-1, Akt-2, and Akt-3, which share an about 80% homology. Akt is a downstream mediator of the RTKs that, after activation, engage PI3K, which in turn phosphorylates phosphatidylinositol-4,5-bisphosphate to convert it to phosphatidylinositol-3,4,5-triphosphate (PIP3). Akt moves to the plasma membrane and binds PIP3, as well as phosphoinositide-dependent kinase-1 (PDK-1), that phosphorylates Akt on threonine 308 [14]. The complete activation of Akt occurs upon phosphorylation of serine 473 by the mammalian target of rapamycin protein kinase (mTOR) [14]. Phosphatase and tensin homolog (PTEN) regulates PIP3 levels removing phosphate from the 3-OH position [16]. Mutations in RTKs and aberrant activation of their intracellular signaling pathways have been causally linked to cancer, diabetes, inflammation, bone disorders, arteriosclerosis, and angiogenesis [10,17,18]. These considerations led to the development of a new generation of drugs that block or attenuate RTK activity [10].

## 4. Akt Signaling and Cancer

Accumulating evidence showed a central role of Akt in cancer development and treatment [19]. Although all Akt isoforms are ubiquitously expressed their level may differ, thus, modulating their biological activities in the same tissue [20]. The most diffusely investigated adult stem cells for their role in cancer progression are the mesenchymal stem/stromal cells (MSCs) [21]. The latter may affect cancer progression through a number of secreted factors triggering activation of various cell signaling pathways [21]. These signals may either result in an increased growth and metastasis or lead to growth inhibition and increase of cell death [21]. Thus, MSCs can play a dual role in cancer progression depending on the cellular context wherein they reside. The PI3K/Akt signaling pathway has been documented to play a central role in regulating tumor growth, and several MSC-secreted factors can stimulate this pathway [21]. In many organs, including stomach, colon, and breast, tumor invasion of surrounding or visceral adipose tissue represents a hallmark of invasive capacity. Moreover, infiltration of perivisceral adipose tissue represents another sign of cancer aggressiveness and ASCs play a critical role in adipose tissue homeostasis.

## 5. Akt Signaling and Adipogenic Differention of ASCs

**Figure 3.**
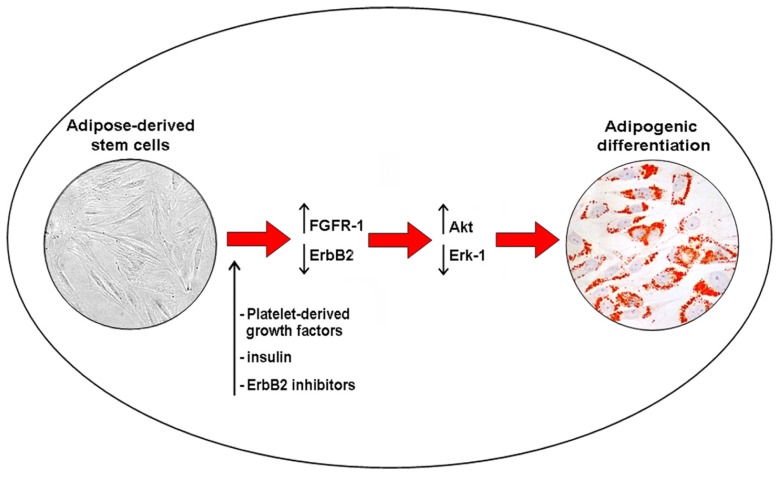
Adipogenic differentiation of ASCs. Schematic representation of adipogenic differentiation of ASCs: FGFR-1 activity and ErbB2 signaling downregulation are involved in the upregulation of Akt pathway and downregulation of Erk-1 in adipogenic differentiation of ASCs.

Various receptor pathways regulate ASC proliferation and differentiation. FGFRs and the ErbB tyrosine kinase receptor family are involved in the control of both growth and differentiation in cancer stem cells and ASCs [3,22–25]. Previous studies documented the presence of EGFR and ErbB2 transcripts and proteins in ASCs [3]. The use of platelet-derived growth factors and hormones, including insulin, stimulated ASC differentiation (Figure 1C,D) and improved the long-term maintenance of fat grafting in patients affected by soft tissue defects [3]. Increased Akt activity plays a crucial role in this process, as well as through the parallel downregulation of EGFR and ErbB2 expression, and Erk-1 activity. Because Erk-1 sustains cell mitotic clonal expansion, it is likely that Erk-1 signal transduction pathway needs to be shut off to allow adipogenic differentiation [26]. In addition, specific inhibition of ErbB2 activity in ASCs increased intracytoplasmic lipid accumulation and upregulated Akt phosphorylation [3]. Akt inhibition was also found to block the differentiation of murine 3T3-L1 preadipocytes [27]. Akt phosphorylation is also needed for the differentiation of murine brown adipocytes and PI3K inhibition blocks the differentiation [28]. A similar result was obtained for ASC adipogenic differentiation after the treatment with wortmannin, a well-known Akt inhibitor [3]. Altogether, these data suggested that ASC adipogenic differentiation involves PI3K activity and its downstream target gene, Akt. Increased adipogenic differentiation of ASCs is also linked to the increase in FGFR-1 and FGFR-2 transcript levels. The selective inhibition of FGFR-1 reduced intracytoplasmic lipid droplet accumulation and Akt phosphorylation of cultured ASCs, suggesting a complex receptor-mediated control (Figure 3) [3]. Moreover, the inhibition of FGFR-1, EGFR and ErbB2 also reduced ASC proliferation [3]. In the breast, the interplay between ASCs and epithelial cells is still partially uninvestigated. Breast adipose tissue is the major source of estrogen delivery [25]. Locally delivered estrogen induces growth and tumorigenesis through the upregulation of various growth factors, including EGFR and Akt activation [25]. A crosstalk between ErbB and estrogen receptor signaling has been reported in both tumor progression and resistance to endocrine therapy of breast cancer cells [29]. Aberrant FGFR activity is also involved in the progression of breast cancer [30], in particular, FGFR-1 upregulation associates with early relapse and poor survival in breast cancer patients [31]. Consequently, at the present, the interplay between resident/grafted ASCs and residual breast cancer cells deserves further investigations, in order to promote specific benefical effects of ASC differentiation during breast reconstruction procedures following cancer surgery.

## 6. Adipose-Derived Stem Cell Angiogenesis and Adipogenesis: Crosstalk and Similarities

**Figure 4.**
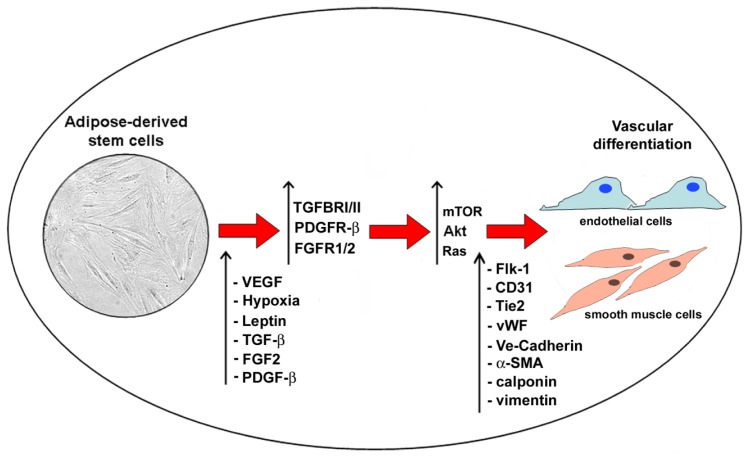
Vascular differentiation of ASCs. Schematic representation of vascular differentiation of ASCs. In particular, under different stimuli (VEGF, leptin, hypoxia, TGF-b, FGF2, PDGF-β), ASC receptor signaling (TGFBRI/II, FGFR1/2, PDGFR-β) is activated and downstream targets upregulated in order to induce vascular differentiation.

Substantial evidence shows that normal tissue growth and regeneration depend on neoangiogenesis [32]. Recent findings support the hypothesis that neovascularization and adipogenesis are chronologically and spatially coupled processes during prenatal life and continue to reciprocally interact via paracrine signaling system throughout the adult life [32]. Activated adipocytes produce multiple angiogenic factors including leptin, angiopoietins, hepatocyte growth factor (HGF), granulocyte-macrophage colony-stimulating factor (GM-CSF), vascular endothelial growth factor (VEGF), basic fibroblast growth factor (FGF-2) and transforming growth factor β (TGF-β), which either alone or collectively stimulate neovascularization during fat mass expansion [32] (Figure 4). Recent studies indicate that ASCs and MSCs are capable to promote neoangiogenesis through the secretion of growth factors, in particular VEGF [33,34]. At the same time, angiogenesis is one crucial event during cancer development and growth [35]. VEGF secretion and interaction with its natural receptors VEGFR-1 and VEGFR-2 play a pivotal role in this process and targeted interferences are crucial for the future strategies for cancer prevention [36,37]. Resident and circulating stem cells contribute to vascular remodelling through collagen synthesis, secretion of growth factors and phenotype changes of vascular cells [38,39]. In particular, ASCs secrete HGF, tumor necrosis factor-α (TNF-α), and nerve growth factor (NGF) [32]. NGF and its precursor proNGF are apoptotic inducers of vascular cells [40]. The exposure of ASCs for 10 days to hypoxia in combination with leptin increased the percentage of CD31^+^ endothelial cells as well as CD31, VE-Cadherin, Flk-1, Tie2, von Willebrand factor, and endothelial cell nitric oxide synthase mRNA [41]. The transcription of these angiogenic markers was further enhanced by co-incubation of VEGF and leptin, and human ASCs cultured on a matrigel surface under hypoxia/VEGF/leptin showed a stable branching network [41]. VEGF/leptin-induced endothelial differentiation of ASCs was dependent on Akt pathway activation, since Akt inhibitor abolished those effects [41]. In addition, ASC genetic modification generating high level of a costitutively active Akt further increased VEGF secretion and the conditioned medium of Akt transfected ASCs injected into a mouse model promoted wound healing compared to conditioned medium from wild type ASCs [42]. Altogether, these findings support the central and critical role of Akt in the regulation of adipogenic and vasculogenic differentiation of ASCs.

## 7. Conclusions

The present review outlines the biomolecular process regulating the adipogenic differentiation of ASCs, that involves RTKs, in particular FGFRs and ErbB receptors, and their downstream effector Akt. Recent studies support the hypothesis that biomolecular pathways regulating adipogenic and vasculogenic differentiation of ASCs are similar and both regulated by complex receptor-mediated pathways. Further studies are needed to provide more information about the regulatory mechanisms involved in the specific differentiative lineages of ASCs, in order to obtain suitable therapeutic instruments for new and safe technologies in the field of reconstructive surgery.

## References

[1] Traktuev D.O., Merfeld-Clauss S., Li J., Kolonin M., Arap W., Pasqualini R., Johnstone B.H., March K.L. (2008). A population of multipotent CD34-positive adipose stromal cells share pericyte and mesenchymal surface markers, reside in a periendothelial location, and stabilize endothelial networks. Circ. Res.

[2] Gimble J.M., Katz A.J., Bunnell B.A. (2007). Adipose-derived stem cells for regenerative medicine. Circ. Res.

[3] Cervelli V., Scioli M.G., Gentile P., Doldo E., Bonanno E., Spagnoli L.G., Orlandi A. (2012). Platelet-rich plasma greatly potentiates insulin-induced adipogenic differentiation of human adipose-derived stem cells through a serine/threonine kinase Akt-dependent mechanism and promotes clinical fat graft maintenance. Stem Cells Transl. Med.

[4] Gentile P., Orlandi A., Scioli M.G., di Pasquali C., Bocchini I., Cervelli V. (2012). Concise review: Adipose-derived stromal vascular fraction cells and platelet-rich plasma: Basic and clinical implications for tissue engineering therapies in regenerative surgery. Stem Cells Transl. Med.

[5] Heng B.C., Liu H., Cao T. (2005). Transplanted human embryonic stem cells as biological “catalysts” for tissue repair and regeneration. Med. Hypotheses.

[6] Spitalieri P., Quitadamo M.C., Orlandi A., Guerra L., Giardina E., Casavola V., Novelli G., Saltini C., Sangiuolo F. (2012). Rescue of murine silica-induced lung injury and fibrosis by human embryonic stem cells. Eur. Respir. J.

[7] Xu Y., Malladi P., Wagner D.R., Longaker M.T. (2005). Adipose-derived mesenchymal cells as a potential cell source for skeletal regeneration. Curr. Opin. Mol. Ther.

[8] Mitchell J.B., McIntosh K., Zvonic S., Garrett S., Floyd Z.E., Kloster A., di Halvorsen Y., Storms R.W., Goh B., Kilroy G. (2006). Immunophenotype of human adipose-derived cells: Temporal changes in stromal-associated and stem cell-associated markers. Stem Cells.

[9] Pittenger M.F., Mackay A.M., Beck S.C., Jaiswal R.K., Douglas R., Mosca J.D., Moorman M.A., Simonetti D.W., Craig S., Marshak D.R. (1999). Multilineage potential of adult human mesenchymal stem cells. Science.

[10] Lemmon M.A., Schlessinger J. (2010). Cell signaling by receptor tyrosine kinases. Cell.

[11] Blume-Jensen P., Hunter T. (2001). Oncogenic kinase signaling. Nature.

[12] Ullrich A., Schlessinger J. (1990). Signal transduction by receptors with tyrosine kinase activity. Cell.

[13] Bafico A., Aaronson S.A., Kufe D.W., Pollock R.E., Weichselbaum R.R. (2003). Signaling pathways of tyrosine kinase receptors. Holland-Frei Cancer Medicine.

[14] Marshall C.J. (1995). Specificity of receptor tyrosine kinase signaling: Transient *versus* sustained extracellular signal-regulated kinase activation. Cell.

[15] Schlessinger J. (1994). SH2/SH3 signaling proteins. Curr. Opin. Genet. Dev.

[16] Zhao M. (2007). PTEN: A promising pharmacological target to enhance epithelial wound healing. Br. J. Pharmacol.

[17] Ciuffreda L., di Sanza C., Cesta Incani U., Eramo A., Desideri M., Biagioni F., Passeri D., Falcone I., Sette G., Bergamo P. (2012). The mitogen-activated protein kinase (MAPK) cascade controls phosphatase and tensin homolog (PTEN) expression through multiple mechanisms. J. Mol. Med. (Berl.).

[18] Trisciuoglio D., de Luca T., Desideri M., Passeri D., Gabellini C., Scarpino S., Liang C., Orlandi A., del Bufalo D. (2013). Removal of the BH4 domain from Bcl-2 protein triggers an autophagic process that impairs tumor growth. Neoplasia.

[19] Wendel H.G., de Stanchina E., Fridman J.S., Malina A., Ray S., Kogan S., Cordon-Cardo C., Pelletier J., Lowe S.W. (2004). Survival signaling by Akt and eIF4E in oncogenesis and cancer therapy. Nature.

[20] Le Page C., Koumakpayi I.H., Alam-Fahmy M., Mes-Masson A.M., Saad F. (2006). Expression and localisation of Akt-1, Akt-2 and Akt-3 correlate with clinical outcome of prostate cancer patients. Br. J. Cancer.

[21] Torsvik A., Bjerkvig R. (2013). Mesenchymal stem cell signaling in cancer progression. Cancer Treat. Rev.

[22] Flågeng M.H., Knappskog S., Haynes B.P., Lønning P.E., Mellgren G. (2013). Inverse regulation of EGFR/HER1 and HER2–4 in normal and malignant human breast tissue. PLoS One.

[23] Nguyen P.T., Tsunematsu T., Yanagisawa S., Kudo Y., Miyauchi M., Kamata N., Takata T. (2013). The FGFR1 inhibitor PD173074 induces mesenchymal-epithelial transition through the transcription factor AP-1. Br. J. Cancer.

[24] Quarto N., Longaker M.T. (2008). Differential expression of specific FGF ligands and receptor isoforms during osteogenic differentiation of mouse Adipose-derived Stem Cells (mASCs) recapitulates the *in vivo* osteogenic pattern. Gene.

[25] Liu B., Ordonez-Ercan D., Fan Z., Huang X., Edgerton S.M., Yang X., Thor A.D. (2009). Estrogenic promotion of ErbB2 tyrosine kinase activity in mammary tumor cells requires activation of ErbB3 signaling. Mol. Cancer Res.

[26] Bost F., Aouadi M., Caron L., Binétruy B. (2005). The role of MAPKs in adipocyte differentiation and obesity. Biochimie.

[27] Zhang H.H., Huang J., Düvel K., Boback B., Wu S., Squillace R.M., Wu C.L., Manning B.D. (2009). Insulin stimulates adipogenesis through the Akt-TSC2-mTORC1 pathway. PLoS One.

[28] Fasshauer M., Klein J., Kriauciunas K.M., Ueki K., Benito M., Kahn C.R. (2001). Essential role of insulin receptor substrate 1 in differentiation of brown adipocytes. Mol. Cell. Biol.

[29] Normanno N., di Maio M., de Maio E., de Luca A., de Matteis A., Giordano A., Perrone F., NCI-Naple Breast Cancer Group (2005). Mechanisms of endocrine resistance and novel therapeutic strategies in breast cancer. Endocr.-Relat. Cancer.

[30] Grose R., Fantl V., Werner S., Chioni A.M., Jarosz M., Rudling R., Cross B., Hart I.R., Dickson C. (2007). The role of fibroblast growth factor receptor 2b in skin homeostasis and cancer development. EMBO J.

[31] Turner N., Pearson A., Sharpe R., Lambros M., Geyer F., Lopez-Garcia M.A., Natrajan R., Marchio C., Iorns E., Mackay A. (2010). FGFR1 amplification drives endocrine therapy resistance and is a therapeutic target in breast cancer. Cancer Res.

[32] Cao Y. (2007). Angiogenesis modulates adipogenesis and obesity. J. Clin. Investig.

[33] Kinnaird T., Stabile E., Burnett M.S., Lee C.W., Barr S., Fuchs S., Epstein S.E. (2004). Marrow-derived stromal cells express genes encoding a broad spectrum of arteriogenic cytokines and promote *in vitro* and *in vivo* arteriogenesis through paracrine mechanisms. Circ. Res.

[34] Selgado A.J., Reis R.L., Sousa N.J., Gimble J.M. (2010). Adipose tissue derived stem cells secretome: Soluble factors and their roles in regenerative medicine. Curr. Stem Cell Res. Ther.

[35] Cesca M., Bizzarro F., Zucchetti M., Gavazzi R. (2013). Tumor delivery of chemotherapy combined with inhibitors of angiogenesis and vascular targeting agents. Front. Oncol.

[36] Tarallo V., Vesci L., Capasso O., Esposito M.T., Riccioni T., Pastore L., Orlandi A., Pisano C., de Falco S. (2010). A placental growth factor variant unable to recognize vascular endothelial growth factor (VEGF) receptor-1 inhibits VEGF-dependent tumor angiogenesis via heterodimerization. Cancer Res.

[37] Cassinelli G., Zuco V., Petrangolini G., de Cesare M., Tortoreto M., Lanzi C., Cominetti D., Zaffaroni N., Orlandi A., Passeri D. (2012). The curative efficacy of namitecan (ST1968) in preclinical models of pediatric sarcoma is associated with antiangiogenic effects. Biochem. Pharmacol.

[38] Orlandi A., Bennett M. (2010). Progenitor cell-derived smooth muscle cells in vascular disease. Biochem. Pharmacol.

[39] Ferlosio A., Arcuri G., Doldo E., Scioli M.G., de Falco S., Spagnoli L.G., Orlandi A. (2012). Age-related increase of stem marker expression influences vascular smooth muscle cell properties. Atherosclerosis.

[40] Campagnolo L., Costanza G., Francesconi A., Arcuri G., Moscatelli I., Orlandi A. (2014). Sortilin expression is essential for pro-nerve growth factor-induced apoptosis of rat vascular smooth muscle cells. PLoS One.

[41] Bekhite M.M., Finkensieper A., Rebhan J., Huse S., Schultze-Mosgau S., Figulla H.R., Sauer H., Wartenberg M. (2014). Hypoxia, leptin, and vascular endothelial growth factor stimulate vascular endothelial cell differentiation of human adipose tissue-derived stem cells. Stem Cells Dev.

[42] Song S.H., Lee M.O., Lee J.S., Jeong H.C., Kim H.G., Kim W.S., Hur M., Cha H.J. (2012). Genetic modification of human adipose-derived stem cells for promoting wound healing. J. Dermatol. Sci.

